# Selection of reference genes for quantitative RT-PCR studies in *Rhipicephalus *(*Boophilus) microplus *and *Rhipicephalus appendiculatus *ticks and determination of the expression profile of Bm86

**DOI:** 10.1186/1471-2199-10-112

**Published:** 2009-12-29

**Authors:** Ard M Nijhof, Jesper A Balk, Milagros Postigo, Frans Jongejan

**Affiliations:** 1 Utrecht Centre for Tick-borne Diseases (UCTD), Faculty of Veterinary Medicine, Utrecht University, Yalelaan 1, 3584 CL, Utrecht, the Netherlands; 2 Department of Tropical Diseases, Faculty of Veterinary Science, University of Pretoria, Private Bag X04, 0110, Onderstepoort, South Africa

## Abstract

**Background:**

For accurate and reliable gene expression analysis, normalization of gene expression data against reference genes is essential. In most studies on ticks where (semi-)quantitative RT-PCR is employed, normalization occurs with a single reference gene, usually β-actin, without validation of its presumed expression stability. The first goal of this study was to evaluate the expression stability of commonly used reference genes in *Rhipicephalus appendiculatus *and *Rhipicephalus (Boophilus) microplus *ticks. To demonstrate the usefulness of these results, an unresolved issue in tick vaccine development was examined. Commercial vaccines against *R. microplus *were developed based on the recombinant antigen Bm86, but despite a high degree of sequence homology, these vaccines are not effective against *R. appendiculatus*. In fact, Bm86-based vaccines give better protection against some tick species with lower Bm86 sequence homology. One possible explanation is the variation in Bm86 expression levels between *R. microplus *and *R. appendiculatus*. The most stable reference genes were therefore used for normalization of the Bm86 expression profile in all life stages of both species to examine whether antigen abundance plays a role in Bm86 vaccine susceptibility.

**Results:**

The transcription levels of nine potential reference genes: β-actin (ACTB), β-tubulin (BTUB), elongation factor 1α (ELF1A), glyceraldehyde 3-phosphate dehydrogenase (GAPDH), glutathione S-transferase (GST), H3 histone family 3A (H3F3A), cyclophilin (PPIA), ribosomal protein L4 (RPL4) and TATA box binding protein (TBP) were measured in all life stages of *R. microplus *and *R. appendiculatus*. ELF1A was found to be the most stable expressed gene in both species following analysis by both geNorm and Normfinder software applications, GST showed the least stability. The expression profile of Bm86 in *R. appendiculatus *and *R. microplus *revealed a more continuous Bm86 antigen abundance in *R. microplus *throughout its one-host life cycle compared to the three-host tick *R. appendiculatus *where large variations were observed between different life stages.

**Conclusion:**

Based on these results, ELF1A can be proposed as an initial reference gene for normalization of quantitative RT-PCR data in whole *R. microplus *and *R. appendiculatus *ticks. The observed differences in Bm86 expression profile between the two species alone can not adequately explain the lack of a Bm86 vaccination effect in *R. appendiculatus*.

## Background

The ixodid ticks *Rhipicephalus appendiculatus *and *Rhipicephalus (Boophilus) microplus *are important pests of livestock. Besides causing direct production losses and leather damage due to their blood-feeding habit, both ticks are able to transmit a wide variety of pathogens. Both tick species overlap in their distribution, but *R. microplus *is more widespread and occurs in subtropical and tropical areas of the world whereas the distribution of *R. appendiculatus*, also known as the brown ear tick, is limited to areas with a humid climate from southern Sudan to the southeastern coast of South Africa. Their life cycle differs quite dramatically too: *R. microplus *is a one-host tick species with all life stages feeding on the same, usually bovine, host whereas *R. appendiculatus *is a three-host tick species with each life stage requiring a new host to feed on. As a consequence of this, *R. microplus *can complete its life cycle in less than 2 months, whereas *R. appendiculatus *takes about 3 months to complete its life cycle under the most favorable conditions [1].

Control of ticks worldwide relies principally on the use of acaricides, but two vaccines targeting *R. microplus *were commercialized in the 1990s: TickGARD Plus^® ^in Australia and Gavac^® ^in Cuba. Both are based on the same recombinant antigen named Bm86, a glycoprotein of unknown function which is located predominantly on the surface of midgut digest cells [2]. Although Bm86-based vaccines showed cross-protection against various other tick species, e.g. *Rhipicephalus (Boophilus) annulatus *[3], *Rhipicephalus (Boophilus) decoloratus*, *Hyalomma anatolicum *and *Hyalomma dromedarii *[4], they were not effective against *Amblyomma variegatum *and *R. appendiculatus *[4,5].

Due to the veterinary and economical importance of *R. microplus *and *R. appendiculatus *in subtropical and tropical areas of the world, expressed sequence tag (EST) datasets for these tick species have been established [6-8]. The availability of these data greatly facilitates research in tick biology and tick-host-pathogen interactions. Microarrays and quantitative RT-PCR are two important techniques measuring gene expression which may help in unraveling such interactions and provide insight into the complex regulatory networks behind biological processes. Output data require normalization to control for variables such as the intrinsic variability of RNA, impurities during RNA extraction, reverse transcription and PCR efficiencies [9]. A frequently used method for the accurate normalization of quantitative RT-PCR data involves the measurement of internal reference genes (also referred to as housekeeping genes). Such genes should ideally have a stable expression independent of cell or tissue type, or experimental condition. A survey of 20 papers using quantitative RT-PCR in tick research published between 2004 and 2008 shows β-actin as the most popular reference gene used for normalization in 19 publications, with the one remaining article employing the 18S rRNA gene. However, the presumed expression stability of these genes in ticks has never been examined and the use of a single reference gene may lead to erroneous normalization [10]. In this study, the mRNA transcript levels of nine commonly used reference genes from different functional classes: β-actin (ACTB), β-tubulin (BTUB), elongation factor 1α (ELF1A), glyceraldehyde 3-phosphate dehydrogenase (GAPDH), glutathione S-transferase (GST), H3 histone family 3A (H3F3A), cyclophilin (PPIA), ribosomal protein L4 (RPL4) and TATA box binding protein (TBP) were measured by quantitative RT-PCR in all life stages of whole *R. microplus *and *R. appendiculatus *ticks. The results were evaluated using geNorm [10] and Normfinder [11]. Although both programs have the same aim of identifying the most stably expressed reference genes, they make use of different strategies. geNorm is a software application which determines the expression stability of reference genes by calculating a gene-stability measure (*M*) for each gene. This measure relies on the principle that the expression ratio of two ideal reference genes is identical in all samples, regardless of the experimental condition or cell type. Pairwise variation for each combination of reference genes is determined and assigned a value for *M*, and genes with the highest *M *value (i.e. least stable expression) are progressively eliminated until the two most stably expressed genes remain. It thus ranks the reference genes according to the similarity in expression profiles across the samples [10]. Incorporation of co-regulated reference genes will affect the outcome of this approach and care must therefore be taken in selecting candidate reference genes from different functional classes. Normfinder is an application on a model-based approach which ranks the reference genes according to the estimated intra- and intergroup expression variation. Normalization with the six most stable expressed reference genes of the Bm86 mRNA transcript levels in all life stages of *R. microplus *and *R. appendiculatus *was carried out with the aim to elucidate the role of antigen abundance in Bm86 vaccine susceptibility.

## Results

### Quantitative RT-PCR

**Figure 1:**
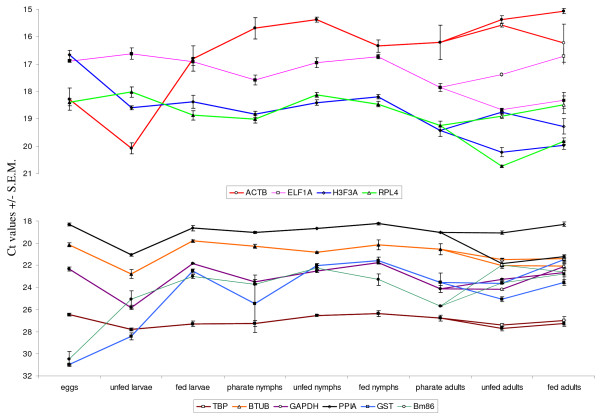
**Bm86 and control gene expression during all life stages of *R. microplus***. Ct values represent mean +/- SEM from three biological replicates. The Ct values of samples from adult females are indicated with an open symbol, Ct values from adult males with a closed symbol. Note that the y-axis differs in the two panels: highly expressed genes are shown in the top panel, moderately expressed genes in the bottom panel.

**Figure 2:**
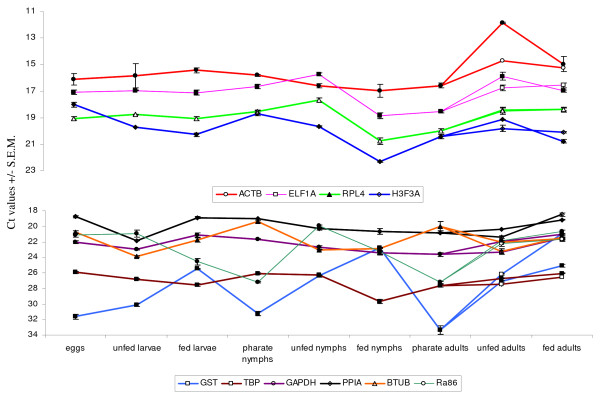
**Ra86 and control gene expression during all life stages of *R. appendiculatus***. Ct values represent mean +/- SEM from three biological replicates. The Ct values of samples from adult females are indicated with an open symbol, Ct values from adult males with a closed symbol. Note that the y-axis differs in the two panels: highly expressed genes are shown in the top panel, moderately expressed genes in the bottom panel.

The efficiencies of the quantitative RT-PCR's were uniformly high and ranged from 91% to 103%, making all assays suitable for quantitative analysis (Table 1). All PCR's generated a single band and the absence of primer dimer formation was confirmed by a dissociation assay performed with each assay (results not shown). None of the primer combinations amplified cDNA synthesized from bovine blood RNA, which excludes interference with the PCR results caused by the possible presence of host RNA in fed ticks. Raw Ct values ranged from 14.8 (ACTB) to 31.0 (GST) in *R. microplus *and from 11.8 (ACTB) to 34.3 (GST) in *R. appendiculatus *(Table 2, Fig. 1 &2). GAPDH, GST and TBP were expressed at low levels in both tick species with median Ct values above 22 cycles. The smallest Ct variation between all samples of *R. microplus *was exhibited by TBP (2.27) and by GAPDH (3.42) in *R. appendiculatus*. GST showed the most variable expression between all samples for both tick species; 10.77 in *R. microplus *and 13.41 in *R. appendiculatus*.

**Table 1:** Details of the quantitative RT-PCRs of Bm86 and the candidate reference genes evaluated in this study.

Symbol	Gene name	Function	GenBank accession number		Forward primer	Reverse primer	Amplicon length	Efficiency	
			*Rm* ^*a*^	*Ra* ^*a*^				*Rm* ^*a*^	*Ra* ^*a*^
ACTB	Beta actin	Cytoskeletal structural protein	AY255624	AY254899	CCCATCTACGAAGGTTACGCC	CGCACGATTTCACGCTCAG	139 bp	102	98
Bm86	Bm86	Unknown	FJ809946	FJ809944	CGTCCCGACTTGACCTGC	AGGAGCGGCTGAACAGTTTG	101 bp	103	103
BTUB	Beta tubulin	Component of microtubules	CK179480	CD781348	AACATGGTGCCCTTCCCACG	GCAGCCATCATGTTCTTTGC	140 bp	92	97
ELF1A	Elongation factor 1-alpha	Component of the eukaryotic translational apparatus	EW679365	CD797149	CGTCTACAAGATTGGTGGCATT	CTCAGTGGTCAGGTTGGCAG	108 bp	100	92
GAPDH	Glyceraldehyde-3-phosphate dehydrogenase	Oxireductase in glycolysis and gluconeogenesis	CK180824	CD791831	AGTCCACCGGCGTCTTCCTCA	GTGTGGTTCACACCCATCACAA	123 bp	97	91
GST	Glutathione S-transferase	Detoxification of endobiotic and xenobiotic substrates	CV456312	CD789942	TACCTGGGCAAGAAGCACGG	AGAGCCCAGAGCAGGTCGTTG	98 bp	93	100
H3F3A	H3 Histone family 3A	Involved in structure of chromatin	CV442167	CD795637	AAGCAGACCGCCCGTAAGT	GTAACGACGGATCTCCCTGAG	152 bp	92	95
PPIA	Cyclophilin	Facilitate protein folding	CV445080	CD793819	CTGGGACGGATAGTAATTGAGC	ATGAAGTTGGGGATGACGC	133 bp	95	98
RPL4	Ribosomal protein L4	Structural component of the large 60S ribosomal subunit	CV447629	CD794864	AGGTTCCCCTGGTGGTGAG	GTTCCTCATCTTTCCCTTGCC	152 bp	93	98
TBP	TATA box binding protein	Transcription factor	CV453818	CD780134	CTTGTCCTCACACACAGCCAGTT	GTGAGCACGACTTTTCCAGATAC	122 bp	94	100

^*a*^Rm, R. microplus; Ra, R. appendiculatus

**Table 2:** Cycle threshold (C_t_) values of candidate reference genes and Bm86

Gene	C_t _Range		**C_t _Min**.		C_t _Max		**mean C_t _± s.e.m**.	
	*Rm* ^*a*^	*Ra* ^*a*^	*Rm* ^*a*^	*Ra* ^*a*^	*Rm* ^*a*^	*Ra* ^*a*^	*Rm* ^*a*^	*Ra* ^*a*^
ACTB	5.62	5.92	14.85	11.80	20.47	17.72	16.48 ± 0.25	15.59 ± 0.24
BTUB	4.01	4.74	19.57	19.22	23.58	23.96	21.00 ± 0.16	21.76 ± 0.24
ELF1A	2.36	3.90	16.36	15.34	18.72	19.24	17.29 ± 0.12	17.01 ± 0.17
GAPDH	4.39	3.47	21.67	20.56	26.06	24.04	23.02 ± 0.20	22.29 ± 0.17
GST	10.77	13.41	20.28	20.90	31.05	34.31	24.27 ± 0.51	27.22 ± 0.67
H3F3A	4.03	4.69	16.35	17.68	20.39	22.36	18.74 ± 0.15	19.90 ± 0.20
PPIA	4.38	3.90	17.92	17.98	22.30	21.88	19.31 ± 0.20	19.91 ± 0.20
RPL4	3.09	3.83	17.68	17.39	20.77	21.22	18.82 ± 0.12	18.87 ± 0.15
TBP	2.27	4.28	25.74	25.70	28.01	29.98	27.01 ± 0.09	26.99 ± 0.20
Bm86	10.38	7.56	21.28	19.86	31.66	27.42	24.05 ± 0.40	22.85 ± 0.44

^*a*^Rm, R. microplus; Ra, R. appendiculatus

### geNorm and Normfinder analysis

The gene expression stability of nine candidate reference genes over the life cycle of *R. microplus *and *R. appendiculatus *was analyzed using the geNorm and Normfinder software applications (Table 3). The geNorm approach identified ELF1A and RPL4 as the best pair of reference genes over the life cycle of both *R. microplus *and *R. appendiculatus*, as well as in a combined analysis of all samples from both species. Normfinder ranked these genes as second and third best in *R. microplus *and *R. appendiculatus *with TBP and GAPDH being indicated as the best reference gene, respectively. A combined analysis of both species by Normfinder ranked ELF1A as best reference gene followed by GAPDH, TBP, PPIA, RPL4, H3F3A, BTUB, ACTB and GST. GST and ACTB were identified as the least stable genes in all groups by both methods.

**Table 3:** Candidate reference genes ranked according to their expression stability as calculated by the Normfinder and geNorm programs.

*R. microplus*		*R. appendiculatus*		*R. microplus *and *R. appendiculatus*	
geNorm	Normfinder	geNorm	Normfinder	geNorm	Normfinder
ELF1A and RPL4 (0.301)	TBP (0.400)	ELF1A and RPL4 (0.371)	GAPDH (0.359)	ELF1A and RPL4 (0.376)	ELF1A (0.477)
	ELF1A (0.459)		ELF1A (0.424)		GAPDH (0.521)
H3F3A (0.559)	RPL4 (0.463)	TBP (0.614)	RPL4 (0.481)	TBP (0.619)	TBP (0.549)
TBP (0.656)	BTUB (0.485)	GAPDH (0.764)	PPIA (0.580)	H3F3A (0.778)	PPIA (0.555)
BTUB (0.771)	PPIA (0.512)	H3F3A (0.856)	TBP (0.583)	PPIA (0.926)	RPL4 (0.563)
PPIA (0.824)	H3F3A (0.577)	PPIA (0.959)	H3F3A (0.709)	GAPDH (0.995)	H3F3A (0.623)
GAPDH (0.940)	GAPDH (0.649)	ACTB (1.097)	ACTB (0.720)	BTUB (1.074)	BTUB (0.680)
ACTB (1.159)	ACTB (0.952)	BTUB (1.203)	BTUB (0.852)	ACTB (1.259)	ACTB (0.962)
GST (1.512)	GST (1.450)	GST (1.818)	GST (2.128)	GST (1.795)	GST (1.899)

The candidates are listed with decreasing expression stability from top to bottom. Average expression stability values are shown between parentheses.

**Figure 3:**
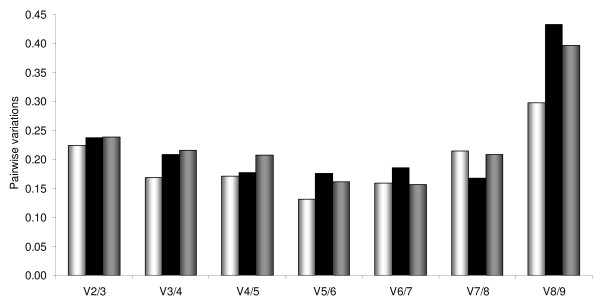
**Optimal number of control genes for normalization as determined by geNorm for *R. microplus *(white bars), *R. appendiculatus *(black bars) and in a combined analysis (grey bars)**.

To determine the minimum number of reference genes necessary for accurate normalization, calculation of the pairwise variation (*V*_*n*/*n*+1_) was performed by geNorm. The lowest *V *values were found to be 0.131 for *V*_5/6 _in *R. microplus *and 0.168 for *V*_7/8 _in *R. appendiculatus*. Combined analysis of both tick species yielded a lowest *V *value of 0.156 at *V*_6/7_(Fig. 3).

### Bm86 and Ra86 sequence analysis

**Figure 4:**
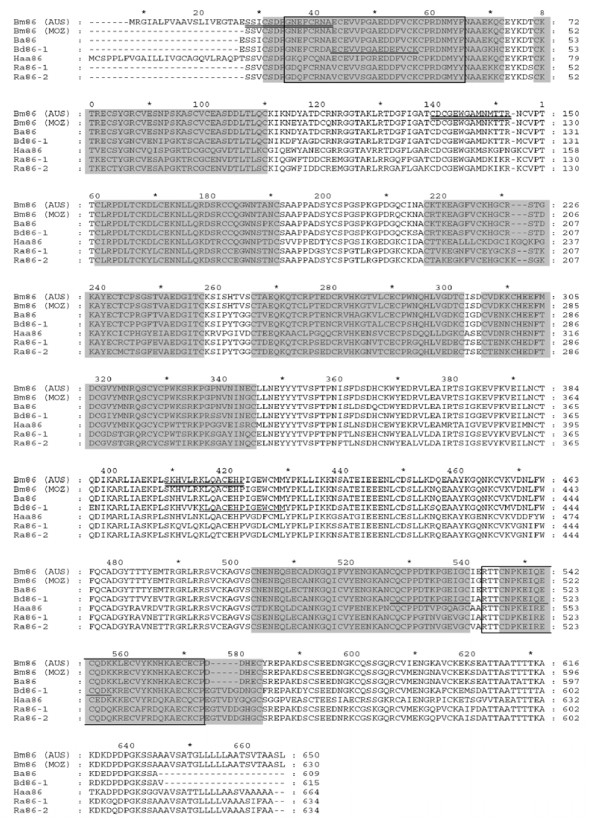
**Alignment of the amino acid sequences of the Bm86 homologues from ticks for which Bm86 vaccine efficiency has been documented: Bm86 AUS (Australian strain) (AAA30098), Bm86 MOZ (Mozambique strain) (FJ809946), Ba86 (ABY58969), Bd86 (ABG21131), Haa86 (AAL36024), Ra86-1 (FJ809944) and Ra86-2 (FJ809945)**. Cross reactive linear B-cell epitopes mapped using pin-coupled peptides by Odongo *et al*. [5] are boxed, the regions identified using biotin-coupled peptides by the same authors are underlined. Three synthetic peptides used by Patarroyo *et al*. [33] which induced an immune response against *R. microplus *are double underlined and EGF-like domains fitting the pattern Cys-Xaa_48_-Cys-Xaa_3-6_-Cys-Xaa_8-11_-Cys-Xaa_0-1_-Cys-Xaa_5-15_-Cys (where Xaa is any amino acid except for cysteine) with 5 or 6 cysteine residues are shaded grey. The phenylalanine at position 507 of Bm86 AUS was predicted to be a cysteine when the sequence of a second cDNA clone from a separate library was determined by Rand *et al*. [12].

**Figure 5:**
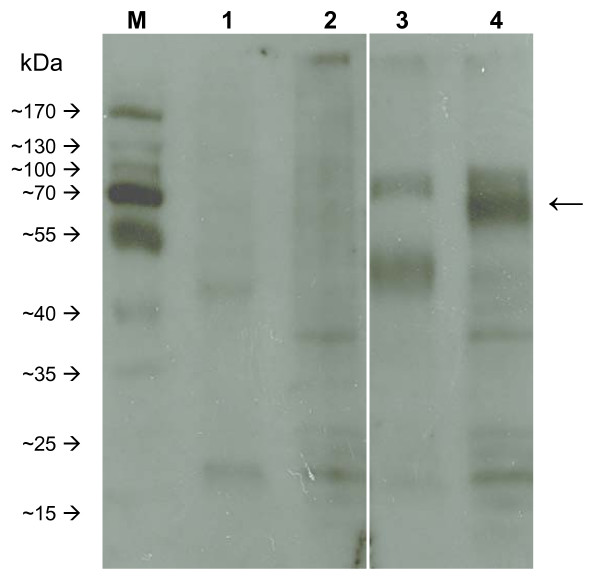
**Immunodetection of Ra86 by Western Blot analysis using ovine Bm86 antiserum**. Lanes 1 and 2: *R. microplus *and *R. appendiculatus *midgut proteins probed with control serum from a sheep vaccinated with adjuvant only, lanes 3 and 4: *R. microplus *and *R. appendiculatus *midgut proteins probed with ovine Bm86 antisera. The arrow on the right indicates the Bm86 and Ra86 proteins.

One sequence for Bm86 (Mozambique) and two sequences from the Bm86 homologue of *R. appendiculatus*, Ra86-1 and Ra86-2, were obtained by 3'RACE with degenerate primer Ra86-F, which is located one amino acid downstream of the signal peptide of Bm86. The open reading frame (ORF) of the Bm86 (Mozambique) nucleotide sequence is 1890 bp, coding for a protein of 629 amino acids which is 96.5% identical to the Bm86 Yeerongpilly reference strain (Australia) with similar structural properties. The 22 amino acid gap reported previously in a second Bm86 (Mozambique) sequence (GenBank accession number ABY58968) was not detected in any of the five sequenced clones. Both *R. appendiculatus *sequences contain a 1905 bp-long ORF which encodes for 634 amino acids. The alleles differ by 45 nucleotides of which 13 are silent mutations and 32 result in a change in the deduced amino acid sequence of the protein [see Additional file 1]. The identity of the amino acid sequence of Ra86-1 and Ra86-2 are 72.9% and 73.8% with the Bm86 proteins of *R. microplus *(Australia) and 73.7% and 74.6% with *R. microplus *(Mozambique) respectively. A comparison between the amino acid sequence of Ra86 and Bm86 shows that Ra86 contains the same Epidermal Growth Factor (EGF)-like domains as Bm86 [12]. These domains are also present in Ba86, Bd86 and Haa86, the Bm86 homologues of *R. annulatus*, *R. decoloratus *and *Hy. a. anatolicum *respectively (Fig. 4). The Ra86 sequences contain 5 potential sites for N-linked glycosylation (Asn-Xaa-Ser/Thr) and Ra86-2 has 1 potential *O*-glycosylation site (Ser/Thr). Both Ra86-1 and Ra86-2 are predicted to contain a glycosylphosphatidylinositol (GPI) modification site at position 613 (serine), which provides linkage of the molecule to the cell membrane. The presence of a GPI anchor is a common feature found in all ixodid tick Bm86 homologues characterized thus far. Western Blot analysis showed that ovine Bm86 antiserum recognized bands of the expected Ra86 protein size in the isolated midguts from partially fed *R. appendiculatus *females whereas serum from a sheep vaccinated with adjuvant only did not (Fig. 5).

### Bm86/Ra86 expression analysis

**Figure 6:**
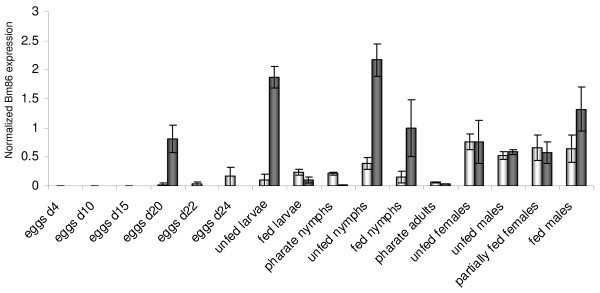
**Bm86 (white bars) and Ra86 (grey bars) expression levels in all life stages, normalized against the six most stably expressed reference genes in both *R. microplus *and *R. appendiculatus*: ELF1A, GAPDH, H3F3A, PPIA, RPL4 and TBP**. Bars represent the 95% confidence interval of the normalized expression. Eggs of *R. appendiculatus *were only collected at day 20 after the start of oviposition and expression levels from other time points of embryogenesis are therefore missing.

The expression profile of Bm86/Ra86 mRNA (referred to as Bm86 from this point onwards for convenience) in both *R. microplus *and *R. appendiculatus *was obtained by normalizing its expression with six reference genes that ranked highest in the geNorm and Normfinder analysis of the combined *R. microplus *and *R. appendiculatus *samples: ELF1A, GAPDH, H3F3A, PPIA, RPL4 and TBP (Fig. 6). In eggs of *R. microplus*, Bm86 expression was detected at low levels in eggs 4 and 10 days after initiation of the oviposition (p.o.: post oviposition) and increased by three-fold in eggs collected 15 days p.o. This formed the start of a rapid increase in the expression of Bm86 in the third trimester of the embryogenesis to levels similar to that found in unfed larvae. Bm86 expression decreased with feeding and molting in the immature life stages, with the lowest expression found in the pharate life stages. The decrease of Bm86 expression levels following feeding of immatures was significantly more pronounced in the larvae and nymphs of *R. appendiculatus *compared to *R. microplus *where a more continuous expression pattern was observed during the life cycle with less dramatic variation. The expression level of Bm86 in adults did not differ significantly between males and females of both species.

## Discussion

To minimize RT-PCR specific errors and correct for sample-to-sample variation in order to make a comparison of the Bm86 expression profiles from *R. microplus *and *R. appendiculatus *possible, appropriate normalization is required. The use of reference genes is most frequently applied to normalize the mRNA fraction, but validation of the expression stability of such genes in ticks has not been reported until now. ACTB is the most commonly used reference gene in tick research, but recent findings in mammals revealed that this gene and other commonly used 'classical' reference genes such as GAPDH may be inappropriate for use as a reference gene because of their variability under experimental conditions [9,13].

The ideal reference gene should be expressed at a constant level in the tissue(s) of interest at all stages of development and be unaffected by the specific experimental treatment being examined. However, no such universal reference gene has yet been identified and probably does not exist [9-11]. Normalization with multiple selected reference genes has been proposed as an alternative to overcome this problem and several tools to evaluate the expression stability of candidate reference genes have been developed. In this study, two of these tools, the geNorm [10] and Normfinder [11] programs, were employed to evaluate the expression stability of nine selected candidate reference genes.

Besides the two 'classical' reference genes ACTB and GAPDH, other candidate reference genes evaluated in this study were from different functional classes and were selected based on their reported expression stability in other organisms and their presence in the EST libraries of *R. microplus *and *R. appendiculatus *[6-8]. RPL4 for instance was among the thirteen ribosomal proteins which were ranked in the top 15 of most stable expressed reference genes in a meta-analysis performed on a large dataset of human gene arrays [13]. Other ribosomal proteins were not included in this study to prevent bias in the ranking of the reference genes due to correlated expression of proteins belonging to the same functional class.

The outcome of the gene stability evaluation differed between the programs used, which is not surprising in light of the different algorithms they employ. Only ELF1A was consistently ranked first or second by both programs and is suitable for use as a reference gene under the conditions described here. RPL4 is consistently ranked as the most stable expressed gene together with ELF1A by geNorm but not by Normfinder. Since both ELF1A and RPL4 play a role in protein translation, co-regulation cannot be ruled out and this may have affected the outcome of the geNorm analysis. Normfinder is less sensitive to the incorporation of co-regulated genes since it focuses on the intra- and intergroup variation in selecting the most stable expressed genes [11]. This may altogether explain the discordance in ranking of RPL4 between the geNorm and Normfinder programs. GST turned out to be the least stable gene in all conducted analyses (Table 3). GST is known to be differentially expressed under different conditions [14-16] and so could be expected to perform poorly as a reference gene. Of the 'traditional' reference genes, ACTB was ranked among the least stable genes by both methods whereas GAPDH was ranked first in the Normfinder analysis of the *R. appendiculatus *life stages. This is a direct result of the small Ct variation observed in the GAPDH expression in this species, which also explains the high ranking of TBP in the Normfinder analysis of the *R. microplus *life stages where this gene showed the least Ct variation over all samples.

Analysis of the pairwise variation (*V*_*n*/*n*+1_) of the samples by geNorm returned values slightly lower or higher than the arbitrarily chosen threshold of 0.15 [10], reflecting the heterogeneous nature of the analyzed whole tick samples which varied from egg to feeding adults. A direct consequence is the need of using a larger number of reference genes for optimal normalization. To be able to compare the Bm86 mRNA expression between all life stages of *R. microplus *and *R. appendiculatus*, normalization with six reference genes: ELF1A, RPL4, TBP, H3F3A, PPIA and GAPDH was conducted. These reference genes were evaluated as being the most stable by both geNorm and Normfinder in a combined analysis of all samples from both tick species and returned the lowest pairwise variation value in the geNorm analysis (*V*_6/7 _= 0.156).

The protein sequence of the Bm86 gene from the Mozambique *R. microplus *strain was highly identical to previously reported Bm86 sequences from Australia and South America and shared a maximum identity of 97.0% with the Bm86 sequence from a *R. microplus *strain from central Brazil (GenBank accession number ACA57829). A second Bm86 sequence isolated from the viscera of partially fed females originating from the same Mozambique tick colony (GenBank accession number ABY58968) contains a 22 amino acid gap which was not present in the Bm86 sequence identified in this study [17]. This sequence was not found in any of the five sequenced clones and may be less abundant, have been the result of alternative splicing or represent an allele which was lost in the *R. microplus *tick population since the source material was collected several generations earlier. The presence of Bm86 alleles within the same tick population has been previously reported [18]. Two alleles, Ra86-1 and Ra86-2, were also found to be transcribed in the midgut of *R. appendiculatus *females. The ORFs were similar in size but the alleles differed by 32 amino acids. These differences do not appear to have a striking effect on the main properties of these proteins since both Ra86-1 and Ra86-2 are predicted to contain a GPI-anchor and contain EGF-like domains similar to those found in Bm86 (Fig. 4), but Ra86-2 does have a single potential *O*-glycosylation site which is absent from Ra86-1. Since *Boophilus *species were recently synonymized with the *Rhipicephalus *genus [19], it is not surprising that the Ra86 protein shows a higher amino acid sequence identity with Bm86 (~73%) compared to the Haa86 protein, the Bm86 homologue from the two-host tick *Hy. anatolicum *(65%). However, feeding of *Hy. anatolicum *on cattle vaccinated with a recombinant Bm86 vaccine does result in a deleterious effect against this tick species which is not seen in *R. appendiculatus *[4]. Since Western Blot analysis showed that ovine Bm86 antisera does indeed recognize *R. appendiculatus *proteins (Fig. 5), other biological factors such as conformational epitopes, amount of blood/antibodies ingested, or antigen abundance may play a role in the biology of Bm86 vaccine susceptibility. To investigate the latter hypothesis, the Bm86 and Ra86 transcript levels were measured throughout the life cycle of both tick species feeding on the same host by quantitative RT-PCR using a single primer pair which amplifies all known alleles of Bm86 and Ra86.

The normalized Bm86 mRNA expression levels were monitored in various stages of embryonic development in *R. microplus *and were found to increase exponentially during the last 9 days prior to hatching, simultaneous with the development of the midgut in embryos which takes place in the third trimester of embryogenesis in ixodid ticks [20,21] (Fig. 6). At day 20 p.o. expression levels of Bm86 were 18 (8-40) times higher in *R. appendiculatus *eggs compared to *R. microplus *eggs, a difference that might in part be explained by a more advanced egg development in *R. appendiculatus *eggs as they were noted to hatch one day earlier than eggs from *R. microplus*. The same large difference in Bm86 expression level was also observed in unfed larvae and nymphs where Bm86 expression levels were approximately tenfold higher in *R. appendiculatus *immatures in anticipation of a blood meal compared to unfed *R. microplus *larvae and nymphs. It should be noted that all samples were collected at single well defined points from each life stage and fluctuations possibly occurring during these life stages could therefore have been missed. The Bm86 expression decreased significantly during feeding and particularly during molting in *R. appendiculatus*, a decrease which was present in *R. microplus *nymphs as well but to a far lesser extent. Bm86 expression levels of adults from both species were similar and so the total amount of Bm86 expressed during blood feeding and exposure to the host immune system may be comparable between adults of the two species, assuming that the expression profile of Bm86 mRNA is indicative for the amount of expressed Bm86 protein. If so, differences in Bm86 vaccination susceptibility could perhaps be sought in the prolonged exposure to imbibed blood and the host immune system of *R. microplus *which is adapted for continuous development on one host compared to the three-host tick *R. appendiculatus*. The latter has a longer 'recovery' period during molting at which time no or very little Bm86 is expressed. Hence little reaction between ingested antibodies and the Bm86 protein would be expected to occur. However, effects of vaccination with Bm86 are predominantly seen in adults of *R. microplus *[22]. This is corroborated by the fact that if *R. microplus *are raised to the stage of unfed adults on non-vaccinated cattle, then transferred to either vaccinated sheep [23] or to an in-vitro feeding system using blood from vaccinated cattle [22], strong vaccine effects are seen. As mentioned earlier, this effect is not seen in *R. appendiculatus *adults feeding on cows vaccinated with Bm86 [4,5], although both *R. microplus *and *R. appendiculatus *have comparable Bm86 expression levels in both unfed and fed adults.

Although the nomenclature used to distinguish the various cell types present in the midgut of ticks is not unanimous, the midgut is thought to consist of the following epithelial cell types: stem cells, also referred to as undifferentiated reserve cell [24] or replacement cell [25], various stages of digest cells, secretory cells and albeit controversial, a basophilic cell type [26-28]. The digest cells are thought to derive from the stem cells and transform from a prodigest cell type to a sessile digest cell following the absorption of blood meal haemoglobin. Sessile cells may detach from the basal lamina into the gut lumen, thus becoming detached or motile digest cells. Upon release of their hematin granules and other indigestible products into the lumen they are termed spent or degenerating digest cells [27]. Exhausted digest cells are replaced by consecutive cycles of growth and differentiation from undifferentiated cells so multiple generations of a digest cell type may be present at the same time, which has led to some confusion in the interpretation of these events [29]. While it has been reported that the Bm86 protein is located predominantly on the microvilli surface of digest cells, the exact cell type could not be determined for technical reasons [2]. The high Bm86 expression levels found in eggs in the third trimester of embryogenesis and unfed larvae suggest that stem cells and/or prodigest cells are expressing Bm86 protein as well. This would be in concordance with the hypothesized function of Bm86 in the regulation of cell growth based on its sequence and structural homology to epidermal growth factor precursors [30] and the greater abundance of Bm86 towards the apical tips of gut digest cells associating it with a regulatory role in the apical growth [2]. Since antibodies from vaccination sera will bind to tick gut cells and inhibit their endocytotic function, involvement of Bm86 in endocytosis of the blood meal seemed possible. However, the inhibition of endocytosis was suggested to be an indirect effect of Bm86 antibodies binding to the Bm86 protein [22]. The low levels of Bm86 expression in feeding and pharate immature ticks and comparable expression levels between unfed and feeding adults make a role for Bm86 in endocytosis more unlikely since the expression of proteins involved in endocytosis is expected to increase during the uptake of a bloodmeal.

## Conclusion

Nine candidate reference genes from different functional classes were identified in the EST databases of *R. microplus *and *R. appendiculatus *and their expression stability throughout the life cycle of these two tick species was evaluated. ELF1A was found to be the most stable expressed gene in both tick species following analysis by both the geNorm and Normfinder software applications, GST showed the least stability. The six most stable expressed genes were used for normalization of the expression profile of the tick-protective antigen Bm86 for both *R. microplus *and *R. appendiculatus*. This expression profile revealed a more continuous Bm86 antigen abundance in *R. microplus *throughout its one-host life cycle compared to the three-host tick *R. appendiculatus *where large variations were observed between the different life stages. The observed differences in Bm86 expression profile between the two species alone can not adequately explain the lack of a Bm86 vaccination effect in *R. appendiculatus*.

## Methods

### Experimental animals

One Holstein-Friesian calf 6 months of age (#1471) was used. The animal had no previous exposure to ticks. All tick feedings were approved by the Animal Experiments Committee (DEC) of the Faculty of Veterinary Medicine, Utrecht University (DEC No. 0111.0807).

### RNA isolation from bovine blood

Total RNA was isolated from 2 ml blood from calf #1471 prior to the tick feedings using the RNeasy mini kit (Qiagen, Venlo, the Netherlands) according to the manufacturer's protocol.

### Ticks and tick feeding

*R. microplus *ticks originating from Mozambique were provided by ClinVet International (Pty), Bloemfontein, South Africa and *R. appendiculatus *ticks originating from South Africa were provided by the Onderstepoort Veterinary Institute, Onderstepoort, South Africa. Both species were subsequently maintained on experimental animals at the tick rearing facility of the Utrecht Centre for Tick-borne Diseases (UCTD) for several generations. Free-living stages were kept at 20°C at 95% relative humidity. Ticks from all life stages of *R. appendiculatus *and unfed larvae of *R. microplus *were available at the start of the experiment. Circular patches used for tick feeding with an inner diameter of 120 mm and sewn to an open cotton bag were glued to the shaved back of the calf using Pattex^® ^contact glue (Henkel Nederland, Nieuwegein, the Netherlands). The scheme shown in Table 4 was used for tick feedings. This schedule allowed for the synchronous feeding of all life stages from both tick species in separate patches on the same animal, minimizing possible variations in tick gene expression due to external (e.g. host or environmental) factors.

**Table 4:** Schedule of tick feeding employed for the synchronous feeding of all life stages from both *R. microplus *and *R. appendiculatus *on calf 1471.

Action	Species	Time point (days)
Collection of eggs	*R. microplus*	4, 10, 15, 20, 22 and 24 days post-oviposition
	*R. appendiculatus*	20 days post-oviposition
Collection of unfed larvae	*R. microplus &* *R. appendiculatus*	21 days post-hatching (45 days post-oviposition)
Placement of unfed larvae	*R. microplus &* *R. appendiculatus*	0
Collection of partially fed larvae	*R. microplus &* *R. appendiculatus*	4
Collection of pharate nymphs	*R. microplus* *R. appendiculatus*	6 7 days post-engorgement
Collection of unfed nymphs	*R. microplus* *R. appendiculatus*	7 21 days post-molting
Placement of unfed nymphs	*R. appendiculatus*	7
Collection of partially fed nymphs	*R. microplus &* *R. appendiculatus*	11
Collection of pharate adults	*R. microplus* *R. appendiculatus*	13 7 days post engorgement
Collection of unfed males	*R. microplus* *R. appendiculatus*	14 21 days post-molting
Collection of unfed females	*R. microplus* *R. appendiculatus*	15 21 days post-molting
Placement of unfed adults	*R. appendiculatus*	15
Collection of partially fed adults	*R. microplus &* *R. appendiculatus*	22

### RNA isolation

For the isolation of total RNA from eggs and unfed larvae, triplicate pools of 100 mg eggs or larvae were homogenized in 1 ml TRIzol reagent using a Potter-Elvejhem glass/Teflon homogenizer. Other whole tick samples were homogenized in 1 ml TRIzol reagent using an ultra-turrax homogenizer (IKA werke GmbH & Co., Staufen, Germany), again in triplicate. All samples were further homogenized by passage through 24- and 27-gauge needles and centrifuged at 12,000 g at 4°C for 10 min to remove insoluble material after which and the supernatant was frozen at -80°C until RNA extraction. Total RNA was isolated and treated with DNase I (Fermentas GmbH, St. Leon Rot, Germany) prior to purification using the Nucleospin RNA II kit (Machery-Nagel, Düren, Germany), all in accordance with the manufacturer's protocols. Sample concentrations and purity were determined with a NanoDrop ND-1000 spectrophotometer (NanoDrop Technologies, Wilmington, DE, USA) at 260 nm (A260) wavelength. Only samples with A260/A280 and A260/A230 ratios between 1.8 and 2.2 were included in subsequent analyses. Lack of genomic DNA contamination was confirmed by PCR amplification of RNA samples followed by electrophoresis on a 1% agarose gel.

### Rapid amplification of 3'cDNA ends (3'-RACE), cloning and sequencing of the Bm86 and Ra86 gene

1 thousand ng of total RNA isolated from the midguts of partially fed *R. microplus *(Mozambique) and *R. appendiculatus *(South Africa) females was used to synthesize first-strand cDNA using SuperScript III (Invitrogen) following the manufacturer's instruction using a 3'-RACE anchor primer containing a poly-T sequence [5'-GCTATCATTACCACAACACTCT_(18)_(AGC)(AGCT)-3']. The Bm86 orthologues were subsequently PCR amplified from this cDNA using degenerate primer Ra86-F [5'-TCATC(CT)(AG)T(CT)TGCTCTGACTTCGG-3'] and a 3'-RACE anchor primer [5'-GCTATCATTACCACAACACTC-3']. The resulting PCR products were purified using the Nucleospin Extract kit (Machery-Nagel), cloned into the pGem-T easy vector (Promega) and five clones of each product were sequenced by Baseclear, Leiden, the Netherlands. Quantitative RT-PCR primers amplifying both Bm86 and Ra86 were designed and synthesized as described above, based upon the available sequences (Table 1). The sequences of Ra86-1, Ra86-2 (South Africa) and Bm86 (Mozambique) have been submitted to GenBank and can be retrieved under accession numbers FJ809944, FJ809945 and FJ809946 respectively. *N*-glycosylation and *O*-glycosylation of the deduced Ra86 protein sequence was predicted by the NetNGlyc 1.0 http://www.cbs.dtu.dk/services/NetNGlyc/ and NetOGlyc 3.1 http://www.cbs.dtu.dk/services/NetOGlyc/ servers of the Center for Biological Sequence Analysis (CBS), Technical University of Denmark. Potential GPI-anchor sites were predicted using the online big-PI predictor tool [31]http://mendel.imp.ac.at/gpi/gpi_server.html.

### Identification of reference genes

Protein sequences from a number of potential candidate reference genes from *Drosophila melanogaster *or *Ceanorhabditis elegans *were used for a tblastn search among the nr and the expressed sequence tag (EST) databases of *R. microplus *and *R. appendiculatus*. The sequences which were found for beta-actin (ACTB), β-tubulin (BTUB), elongation factor 1α (ELF1A), glyceraldehyde 3-phosphate dehydrogenase (GAPDH), glutathione S-transferase (GST), H3 histone family 3A (H3F3A), cyclophilin (PPIA), ribosomal protein L4 (RPL4) and TATA box binding protein (TBP) were subsequently aligned using ClustalW http://www.ebi.ac.uk/clustalw/, generated in BioEdit http://www.mbio.ncsu.edu/BioEdit/bioedit.html. Non-degenerate primers were designed using the NetPrimer software application http://www.premierbiosoft.com/netprimer/ and synthesized by Isogen Life Science, IJsselstein, the Netherlands. Accession numbers and main function of each evaluated reference gene are shown in Table 1.

### Gene expression analysis

cDNA was synthesized from 500 ng of DNA-free RNA isolated from bovine blood and all consecutive life stages of *R. microplus *and *R. appendiculatus*: eggs, unfed larvae, feeding larvae, pharate nymphs, unfed nymphs, feeding nymphs, pharate adults, unfed males/females and feeding males/females using the iScript cDNA synthesis kit (Bio-Rad, Veenendaal, the Netherlands) according to the manufacturer's directions and stored at -20°C until use in quantitative RT-PCR. A quantitative RT-PCR assay using SYBR^® ^green detection was designed and optimized for the transcription profiling of nine commonly used reference genes (Table 1). Real-time analysis was carried out on an iCycler thermal cycler (Bio-Rad). RT-PCR amplification mixtures (25 μl) contained cDNA generated from 5 ng of RNA template, 12.5 μl iQ SYBR green Supermix (Bio-Rad) and 400 nM forward and reverse primer. The cycling conditions comprised a 5 min denaturation and polymerase activation step at 95°C, 40 cycles of 95°C for 10 s, 60°C for 30 s and 72°C for 30 s. Upon completion of the amplification program, a dissociation analysis (52°C-95°C) was performed to determine the purity of the PCR amplicons. To estimate amplification efficiencies, a standard curve was generated for each primer pair based on known quantities of cDNA for both *R. microplus *and *R. appendiculatus *(10-fold serial dilutions corresponding to cDNA transcribed from 50 ng to 0.05 ng of total RNA in triplicate) and analyzed using the iQ 5 software (Bio-Rad). All assays included this standard curve, a no-template control and each of the test cDNAs. Primers, amplicon lengths and PCR efficiencies are indicated in Table 1. Raw Ct values were transformed to quantities using the comparative Ct method and specific PCR efficiencies. These quantities were converted to an input file format suitable for subsequent analysis by the geNorm or Normfinder Excel applications which were downloaded from http://medgen.ugent.be/~jvdesomp/genorm/ and http://www.mdl.dk/publicationsnormfinder.htm respectively. Only the egg samples collected at day 20 from *R. appendiculatus *and *R. microplus *were included in the selection of reference genes using geNorm and Normfinder. The Bm86 expression was measured on the same cDNA samples as used for the reference gene analysis but included additional *R. microplus *egg samples collected at days 4, 10, 15, 22 and 24 post oviposition. The Bm86 expression levels were normalized using the geometric mean of selected reference gene quantities in Microsoft Excel following the guidelines described in the geNorm manual [32] and the 95% confidence interval was calculated. Differential gene expression was considered significant when the 95% confidence interval of the mean normalized expression levels did not overlap (equivalent to P < 0.05).

### Protein isolation and Western Blot

Midguts from partially fed females were dissected in a drop of ice-cold phosphate buffered saline (PBS) using a sterile scalpel and watchmaker forceps under a stereo microscope. The midguts of three females from each species were pooled in a tube with 1 ml washing buffer [10 mM Tris, pH 7.4; 10 mM NaCl; 1 × complete mini protease inhibitor cocktail (Roche Applied Science, Almere, the Netherlands)] and homogenized by passing the tissues through a 22G needle coupled to a 2 ml syringe followed by a similar passage using a 27G needle. The homogenized samples were then centrifuged at 4°C for 30 min at 15,000 *g*, followed by a similar second wash. The final pellet was resuspended in 200 μl of a sample buffer [62,5 mM Tris-HCl, pH 6.8; 2% SDS; 10% glycerol; 1 × complete mini protease inhibitor (Roche Applied Science)] and boiled at 100°C for 5 min. The suspension was centrifuged as described above and the protein concentration of the supernatant was measured using a Pierce BCA protein assay kit (Thermo Fisher Scientific, Etten-Leur, the Netherlands) and the Nanodrop ND-1000 spectrophotometer. Five μg of midgut proteins were separated on a 10% SDS-PAGE gel and transferred electrophoretically onto Hybond C nitrocellulose membranes (GE Healthcare, Diegem, Belgium). The membranes were blocked overnight at 4°C with 2% fish gelatin (Sigma-Aldrich, Zwijndrecht, the Netherlands) in Tris-buffered saline Tween 20 buffer (TBST; 20 mM Tris HCl, 0.9% NaCl and 0.05% Tween 20) and washed at room temperature (RT) for 3 × 5 min in TBST buffer. The membranes were subsequently incubated with ovine Bm86 or control antisera diluted 1:2500 in TBST buffer for 1 h at RT followed by 3 × 5 min washing with TBST. Incubation with secondary rabbit antiserum to sheep IgG conjugated with horseradish peroxidase (Nordic Immunology, Tilburg, the Netherlands) diluted 1: 25000 for 1 h at RT followed by a third washing step with TBST for 3 × 12 min at RT was done prior to 2 min incubation with ECL detection reagent (GE Healthcare) and exposure to Hyperfilm ECL (GE Healthcare).

## Abbreviations

ACTB: β-actin; BTUB: β-tubulin; EGF: Epidermal Growth Factor; ELF1A: elongation factor 1α; EST: expressed sequence tag; GAPDH: glyceraldehyde 3-phosphate dehydrogenase; GPI: glycosylphosphatidylinositol; GST: Glutathione S-transferase; H3F3A: H3 histone family 3A; ORF: Open Reading Frame; PBS: Phosphate Buffered Saline; PPIA: cyclophilin; p.o.: post oviposition; RPL4: ribosomal protein L4; RT: room temperature; TBP: TATA box binding protein; TBST: Tris-buffered saline Tween 20.

## Authors' contributions

AMN conceived and performed the experiment and drafted the manuscript. JB and MP assisted in the protein isolations, Western Blots and cloning and sequencing of Ra86. FJ supervised the study and helped to draft the manuscript. All authors read and approved the final version of the manuscript.

## Supplementary Material

Additional file 1 **Additional figure 1**. Nucleotide sequence and derived amino acid sequence of the two sequenced Ra86 alleles. Click here for file
