# Supplementary material for: Selection of reference genes for quantitative RT-PCR studies in Rhipicephalus (Boophilus) microplus and Rhipicephalus appendiculatus ticks and determination of the expression profile of Bm86
Source: BMC Mol Biol. 2009 Dec 29;10:112. doi: 10.1186/1471-2199-10-112 (PMC2809063; doi:10.1186/1471-2199-10-112)
Supplement: Additional file 1 — Additional figure 1. Nucleotide sequence and derived amino acid sequence of the two sequenced Ra86 alleles. [file 1471-2199-10-112-S1.DOC]

1 TCA TCT GTT TGC TCT GAC TTC GGG GAC CAG TTT TGT CGC AAC GCT GTA TGC GAA GTG GTA 60

1 S S V C S D F G D H F C R N A V C E V V 20

61 TCT GGT GCG GAG GAC GAT TTC GTA TGC AAA TGT CCG CGA GAT GGC ATG TAC TAC AAT GCG 120

21 S G A E D D F V C K C P R D G M Y Y N A 40

121 GCA GAA AAA AAA TGC GAA TAT AAA GAT TC^T^_G_ TGC AAA ACA AAA GAG TGC ACC TAT GGA AGA 180

41 A E K K C E Y K D S C K T K E C T Y G R 60

181 TGC GTT GAA AGT GCT CCT GGC AAA ACC CGC TGT GGC TGC GAA AAC GTG GAC ACT CTA ACG 240

61 C V E S A P G K T R C G C E N V D T L T 80 241 CTA AGT TGC AAG ATT CAA GGT TGG TTC A^C^_T_T GAT GAC TGC CGA GAA ATG GGT GGA ACT GCT 300

81 L S C K I Q G W F ^T^_I_ D D C R E M G G T A 100

301 AGG TTG CGC AGA GGT ^C^_G_^A^_C_G TTT C^C^_T_T GGC GCA A^C^_A_^C^_G_ TGC GAC TGT GGC GAA TGG GG^C^_T_ GCA ATG 360

86 R L R R G ^Q^_A_ F ^P^_L_ G A ^T^_K_ C D C D E W G A M 120

361 GAC AAG ATC AAA CGC AA^A^_G_ TGT GCT CCT ACC ACA TGT CTA CGT CCC GAC TTG ACC TGC AAA 420

121 D K I K R K C V P T T C L R P D L T C K 140

421 TAC CTC TGC GAG AAC AAC CTG CTT CAA A^G^_A_G GAT TCT CGT TGC TGC GAG GGA TGG AAC TCT 480

136 Y L C E N N L L Q ^R^_K_ D S R C C E G W N S 160

481 ACA AAC TGT TCA GCC GCT CCT CCA G^A^_C_T G^G^_A_C AGT TAT TGC TCT CCT GGT ACC CTC AGG GGA 540

151 T N C S A A P P ^D^_A_ ^G^_D_ S Y C S P G T L R G 180

541 CCG GAC GGA AAG TGT AAA GAT GCT TGC AC^T^_C_ G^T^_C_G AAA GAA GGC AA^C^_G_ TTT GTC TGC GAG ^T^_C_AT 600

181 P D G K C K D A C T ^V^_A_ K E G ^N^_K_ F V C E ^Y^_H_ 200 601 GGA TGC AAG A^G^_A_^C^_G_ TCA ^A^_G_^C^_G_C AAG AAG GCG TAC GAG TGC A^G^_T_G TGC ACA ^C^_T_CT GGC TTT GAG GTC 660

196 G C K ^S^_K_ S ^T^_G_ K K A Y E C ^R^_M_ C T ^P^_S_ G F E V 220

661 GCT GAA GAT GGC AT^C^_T_ ACC TGC AAA AGT ATT CCG TAC ACA GGC GGC TGC ACT GAC GAG CAA 720

211 A E D G I T C K S I P Y T G G C T D E Q 240

721 AAA CAG ACT TGC CGC CCA AGC GAA GAC TGC CG^C^_T_ GTG CAC AAA GGA AAT GTG ACG TGC GAG 780

241 K Q T C R P S E D C R V H K G N V T C E 260

781 TGC CCG AGG GGT CAG CAT CTG GTG GAG GAT GAG TGC ACA AGT GAA TGC ACG GAG AAC AAA 840

261 C P R G Q H L V E D E C T S E C T E N K 280

841 TGC CAC GAA GAC TTT ACG GAC TGT GGC G^A^_T_C TCT ACG GGC CGG CAA AGA TGC TAT TGT ^C^_T_CA 900

281 C H E D F T D C G ^D^_V_ S T G R Q R C E C ^P^_S_ 300

901 TGG ACA ^T^_A_^C^_T_A AGG AAG CCT AAG TCG GGA GCC TAC ATC AAC CAA TGC GAA CTG AAT GAG TAT 960

301 W T ^S^_I_ R K P K S G A Y I N Q C E L N E Y 320

961 TAC TAC ACG GTG ^C^_T_CA TTC ACT CCA AAC TTC ACA CTC AAT TCT GA^G^_C_ CAT TGC AAC TGG TAC 1020

321 Y Y T V ^P^_S_ F T P N F T L N S ^E^_D_ H C N W Y 340

1021 GAG GCC CTT GTT ^C^_T_TG GAC GCG ATA AGG ACC AGT AT^C^_T_ GGC AGC GAA GTT TAT AAA GTT GAG 1080

341 E A L V L D A I R T S I G S E V Y K V E 360

1081 ^G^_A_TA CTG AAC TGC ACG CAG GA^C^_T_ ATT AAG GCA AGG CTG ATA GCA TCG AAA CCG CTA TCA AAG 1140

361 ^V^_I_ L N C T Q D I K A R L I A S K P L S K 380

1141 CA^A^_C_ GTG CTC CAG AAG CTT CAA ^G^_A_CA TGC GAG CAT CCC GTC GGG GAC TTG TGC ATG CTG TAT 1200

381 ^Q^_H_ V L Q K L Q ^A^_T_ C E H P V G D L C M L Y 400

1201 CCG AAG TTG CCG ATC AAG AAA A^G^_A_C TCT GCG ACA GAA ATT GAA GAA GAG AAC CTT TGC GAC 1260

401 P K L P I K K ^S^_N_ S A T E I E E E N L C D 420

1261 AGC CTC CTC AAG CGT CAG GAA GCT GCC TAC AAG GGT CAG AAC AAA TGC GTC AAG GTC GGT 1320

421 S L L K R Q E A A Y K G Q N K C V K V G 440

1321 AAC ATT TTC TGG TTC CAG TGC GCT GAT GGT TAC AGA GCA GTT AAC GAG ACC ACA CGA GGT 1380

441 N I F W F Q C A D G Y R A V N E T T R G 460

1381 CGC CTA CGC CGC TCC GTG TGC GAA GCT GGA GTT TCT TGC AGC GAA AAT GAA CAG TTG GAG 1440 461 R L R R S V C E A G V S C R E N E Q L E 480

1441 TGT GCC AAT AAA GGA CAA ATA TGT GTC TAC GAA GAA AAC AAA GCG AAT TGC CAG TGT CCA 1500

481 C A N K G Q I C V Y E E N K A N C Q C P 500

1501 CCA GGC ACT AAC GTT GGA GAA GTT GG^T^_C_ TGC ^A^_G_TT GCC CGT ACC ACA TGC GAC CCT AAA GAG 1560

501 P G T N V G E V G C ^I^_V_ A R T T C D P K E 520

1561 ATA CGA GAA TGC CAG GAT CAG AAG CGG GAG TGC G^C^_T_C TTT AGA GAC CAG AAA GCA GAA TGC 1620

521 I R E C Q D Q K R E C ^A^_V_ F R D Q K A E C 540

1621 AAG TGT CCT GAG GGC ACT GTT GAT GAT GGT CAC GGA TGT TCT CG^A^_G_ GAG CCT GCA AAA GAC 1680

541 K C P E G T V D D G H G C S R E P A K D 560

1681 TCT TGC AGC GAA GAG GAC AAT CGT AAA TGT GGA AGC AAA GGG CAG CGT TGT GTA ATG GAA 1740

561 S C S E E D N R K C G S K G Q R C V M E 580

1741 AAA GGA CAG CCT GTT TGT AAA GCA ATA T^T^_C_T GAC GCA ACA ACA GCA GCG ACT ACA ACA ACG 1800

581 K G Q P V C K A I ^F^_S_ D A T T A A T T T T 600

1801 AAA GCG AAA GAC AA^G^_A_ G^G^_A_T CAA GAT CCT GGA AAG TCG AGT GCT GCC GCA GT^T^_C_ TCA GCT ACA 1860 601 K A K D K ^G^_D_ Q D P G K S S A A A V S A T 620

1861 GGG CTC TTG TTG CTG GTC GCA GCT GCT TCA ATT TTC GCC GCA TAG 1905

621 G L L L L V A A A S I F A A End 635
